# Cincinnati Dental Association

**Published:** 1860-08

**Authors:** 


### Proceedings of Societies.

### CINCINNATI DENTAL ASSOCIATION.

A stated meeting of the Society was held at the office of Drs. Wardle & Doughty on Tuesday evening, July 10, 1860.

*Members present*—Drs. Taft, Wardle, Davenport, H. A. Smith, Cameron, H. R. Smith, and Foote.

Minutes of previous meeting read and approved.

Dr. MERIT WELLS was unanimously elected a member of the Society.

No essay was read.

The subject for discussion, viz: Filling Teeth, was then taken up.

Dr. TAFT remarked that a writer in an Eastern Journal had recently, in an article on the subject of filling teeth over a slight exposure of nerve, or where there is a thin covering of bone to protect the pulp, condemned the practice, and he wished to ascertain the practice of the members present in such cases. Do you not frequently fill teeth in this condition with success? Dr. T. said he had for the past twelve years practiced filling over slightly exposed nerves, and had not, he thought, lost more than five per cent. of such cases. He did not fill over a nerve, where there was evidence of inflammation, without first treating the pulp, and in many cases, where he could control the patient, first fill the cavity temporarily. He expressed himself decidedly of the opinion that in a majority of cases the pulp would elaborate a bony material, and protect itself, if properly shielded from irritating agents. He occasionally failed to preserve teeth in a living condition by this method of practice, but did not think this a sufficient reason why he should abandon the practice. It was an injury to the profession and ourselves to do so. Physicians do not cease to give the same remedies in like cases, because a patient occasionally has died under the treatment.

Dr. H. R. SMITH's practice is, if the pulp was slightly exposed, to make an application of creosote and chloride of zinc. He merely wished to cauterize the surface of the pulp, and remarked that he succeeded much better by letting this remain but a short time than when the application was allowed to remain for several hours. Where the pulp was protected by a thin layer of bone, he invariably filled with gold. In consolidating the plug, he avoided any direct pressure over the pulp cavity. Any considerable pressure on the nerve would produce inflammation, and the loss of the vitality of the tooth would result.

Dr. WARDLE said he did not usually succeed so well in cases in which he treated the pulp previous to filling, and referred to several instances in his practice where he had failed, attributing it entirely to the previous application of creosote, tannin and chloride of zinc. He had recently used nitric acid, and though with better results than in the former plan of treatment.

Dr. H. A. SMITH remarked that he had practiced filling with gold where there was but a thin lamina of bone covering the pulp ; but of late, when he did it, did not feel so confident of success as formerly. He had observed numbers of teeth which he filled in this condition had lost their vitality, and were a source of much annoyance to the patient. This did not occur in some instances until a considerable length of time had elapsed after filling. With some individuals there was not the slightest danger of irritation from the near proximity of a metal to the pulp. Persons of good health, with an absence of the inflammatory diathesis, were of this class. We did not, he thought, observe the temperament and idiosyncrasies of our patients carefully enough, and was confident, if more regard was given to this particular, the particular mode of practice now being considered would occur much less frequently.

If the nerve was actually exposed, he was not in ths habit of filling immediately with a metal. If no inflammatory symptoms were present, would introduce a soft non-metallic filling. This he let remain until the nerve had protected itself. The length of time required for this was very variable, and indeed might never take place. It had been remarked by one that he only introduced soft fillings in cases where he could control the individual; but he (Dr. S.) preferred doing it, although he never expected to see the patient again. He had no faith in arching fillings over an exposed pulp, described by members present. The arch was an imaginary one in some cases, he thought. In a certain class of cavities, it would not be difficult to arch in a pulp, but that particular kind of cavities rarely presented themselves. No one admitted they left a vacuum between the bottom of the cavity and the filling, and he could not quite understand how an operator ascertained the amount of pressure actually applied over that part. A covering of some non-conductor over the part he preferred to the uncertainty of an arched filling.

Dr. Foote stated that he filled teeth with exposed nerves, and seldom lost one. He usually first introduced a soft filling, letting it remain for two or three months. This would afford a protection to the part until ossification had begun. It was a provision of nature that the little tubuli became ossified in advance of caries. It will be perceived, if an examination is made with a microscope of a carious tooth, that ossification of the tubuli is taking place in advance of the decay.

If this be true, the propriety of introducing a soft filling over the thin covering of dentine until ossification ensues, would be apparent. If the pulp was wounded, it would heal up in twenty-four hours with the right treatment, excluding the air. It was not different, and was subject to the same laws as an incised wound in any other soft tissue. If the patient was not under control, he would arch up his filling. This could be readily done with crystal gold, by introducing the first portions in the angles and sides of the cavity, bringing it together on the center. Would prefer crystal gold next the walls of the cavity, as the crystals more readily adapted themselves to the irregularities of the sides than foil.

Dr. CAMERON was not in great favor with the practice of filling over exposed nerves, or even where a thin partition of bone only remained. He mentioned a case in his practice where the nerves were not exposed, and after being filled for three months, alveolar abscess resulted. A slight cold he thought the predisposing cause in these cases. Destroying the nerve and filling the fangs he thought the better practice in cases referred to. The application of nitric acid to exposed nerves he had discontinued.

Dr. DAVENPORT said it was his practice to fill where there was a thin lamina of bone over the pulp. He had never filled over an exposed nerve, but should do so when cases offered themselves.

Inquiry was made how members were succeeding with the use of the mallet.

Dr. WARDLE remarked that he liked it better than his patients; that they in many cases objected to it most decidedly.

Dr. TAFT said the majority of his patients with whom he had tried it, preferred the mallet to the hand pressure. He did not think the patient was much inconvenienced by it; he had one the other day with whom he used the mallet for an hour and a half, and the man slept one hour of the time. The mallet, skillfully used, gave a sense of security, inasmuch as the filling was firm and certain. Now when the instrument once slips in the hand of an operator, and pierces or wounds the gums of the patient, that patient is ever after nervous, and feels insecure during an operation; whereas with the mallet, no such accident can occur, and if the instrument is not properly applied, it slips but an eighth of an inch at the most, and no harm can come to the patient. The blows of the assistant could soon be regulated by the motions of the operator's head, with great certainty and precision.

Dr. TAFT reported a case of sensitive dentine which he had treated recently by burnishing the dentine within the cavity.

The tooth had been filled, and the variations of temperature conveyed by the plug caused so great pain that it was found necessary to remove it. At first, so great was the sensitiveness, that simple contact of the instrument gave intense pain ; but by continuing the pressure for a little time, the sensibility was allayed. The cavity was then refilled, and the tooth had given the patient no further trouble. He mentioned another case where a tooth had been filled for three months, at the expiration of which a slight periosteal inflammation was set up. The plug was removed, and the pulp immediately extirpated, and in fifteen minutes the soreness was entirely gone. Thought if arsenious acid had been used to destroy the pulp, a permanent periosteal difficulty would have been the result.

Society adjourned to meet at the office of Drs. Bonsall & Smith, the second Tuesday in September.

